# Cerebral Venous Sinus Thrombosis: A Complication of Secondary Polycythemia From Testosterone Use

**DOI:** 10.7759/cureus.85789

**Published:** 2025-06-11

**Authors:** Manogna Pendyala, Dhayananth Rattaipalivalasu Saravanan, Purnoor Kaur, Vijay K Doddapaneni, Brandon M Wong

**Affiliations:** 1 Department of Internal Medicine, St. Vincent Medical Center, Toledo, USA

**Keywords:** anticoagulation, erythrocytosis, erythropoiesis, polycythemia, seizure, subarachnoid hemorrhage

## Abstract

Cerebral venous sinus thrombosis (CVST) is a rare but potentially life-threatening cerebrovascular disorder often associated with hypercoagulable states. Testosterone use has been associated with erythrocytosis and increased venous thromboembolism risk. We report the case of a 49-year-old male with a history of chronic testosterone use who presented with a new-onset seizure and was subsequently diagnosed with CVST accompanied by a small parietal subarachnoid hemorrhage (SAH). This case highlights the thrombotic complications of testosterone-induced polycythemia and underscores the importance of surveillance, early recognition, and management.

## Introduction

Testosterone therapy, commonly used for hypogonadism and anabolic purposes, has been associated with erythrocytosis, a recognized risk factor for venous thromboembolism, including cerebral venous sinus thrombosis (CVST) [1,2]. Cerebral venous sinus thrombosis is an uncommon but potentially life-threatening condition and is characterized by thrombosis of the dural venous sinuses. This condition leads to an increase in intracranial pressure and hemorrhagic complications, such as subarachnoid hemorrhage in rare cases. Among the numerous risk factors for CVST, hematologic disorders such as polycythemia have been recognized as essential contributors [3]. Polycythemia increases blood viscosity, thereby predisposing to venous thrombosis.

Testosterone therapy has been associated with secondary polycythemia. The different mechanisms through which testosterone causes polycythemia include stimulating erythropoietin production and decreasing hepcidin production, promoting erythropoiesis, and increasing iron availability [4]. While venous thromboembolic events such as deep vein thrombosis (DVT) and pulmonary embolism (PE) are more frequently reported, testosterone-associated CVST is underrecognized [1]. This case highlights the importance of recognizing CVST as a potential complication of secondary polycythemia due to testosterone use, as well as its management.

## Case presentation

A 49-year-old man presented to the emergency department of a rural hospital with a new-onset seizure preceded by a headache and right lower extremity weakness. The seizure was witnessed and described as rhythmic shaking of the right leg. He had a medical history of hypertension and hypothyroidism managed with levothyroxine. He referred to chronic exogenous testosterone use for approximately 20 years for non-therapeutic, anabolic purposes. He denied any history of similar events, recent head trauma, smoking, or alcohol use.

**Figure 1:**
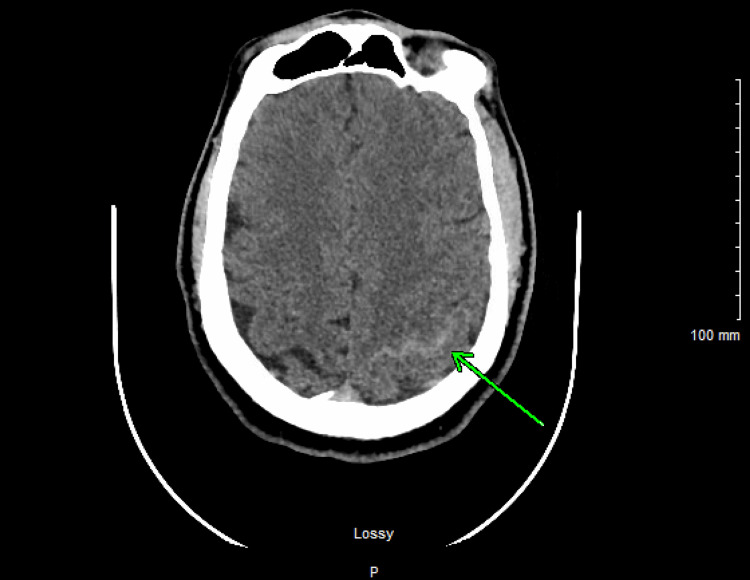
CT of the head demonstrating left-sided subarachnoid hemorrhage (green arrow)

On initial evaluation at the outside facility, he was somnolent, afebrile, hemodynamically stable, and without respiratory distress. He received a dose of intravenous lorazepam 2 mg. Given the leukocytosis, he was given a dose of intravenous ceftriaxone, vancomycin, and dexamethasone. A non-contrast CT obtained at the outside hospital showed a small left parietal-occipital subarachnoid hemorrhage (SAH) (Figure 1). He was then transferred to our hospital for further management.

**Figure 2:**
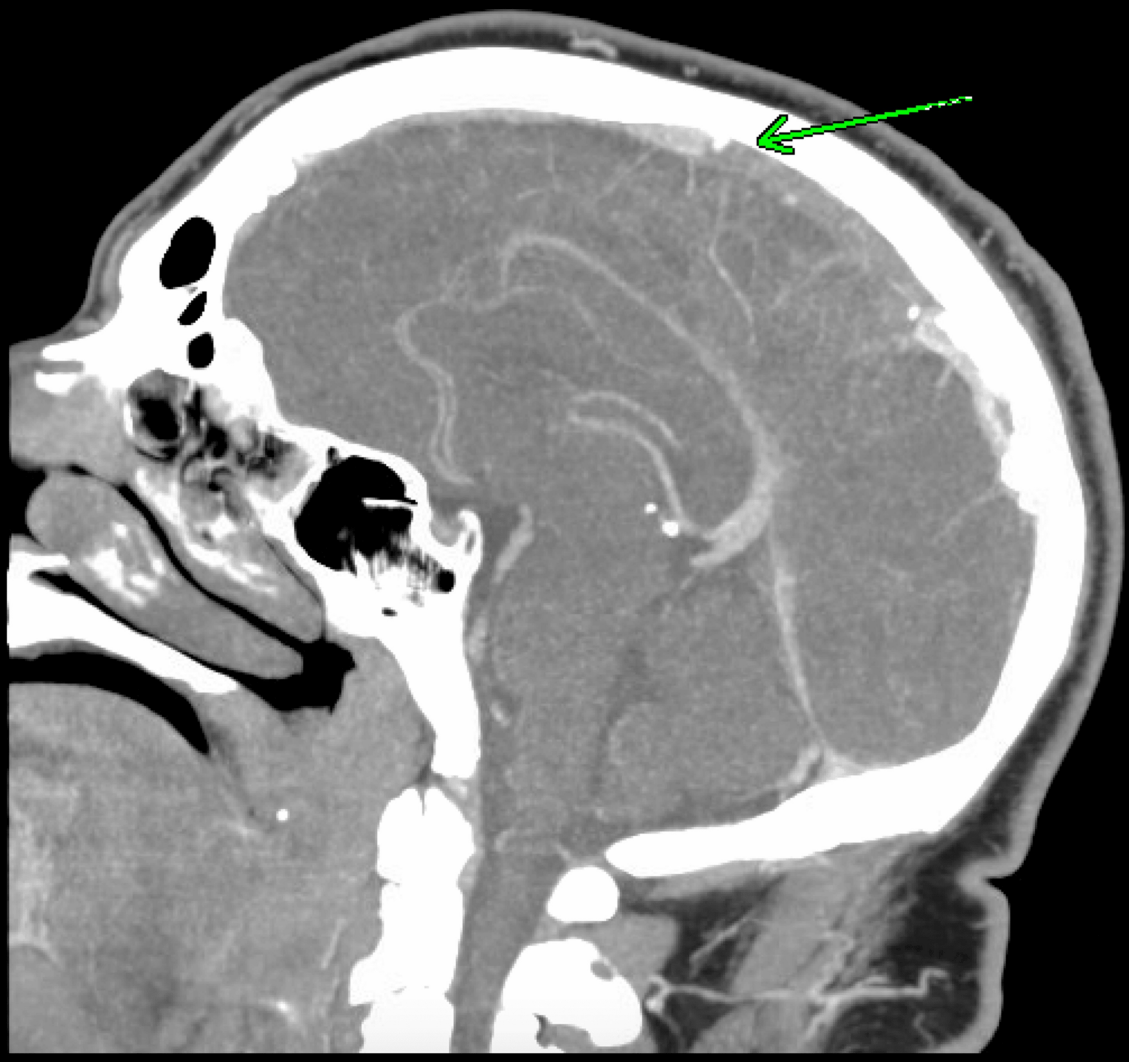
CT venogram of the head and neck showing thrombosis of the superior sagittal sinus (green arrow)

On admission to our hospital, he was alert and oriented, hemodynamically stable, and in no acute distress. On examination, he didn't have any focal deficits. Initial laboratory evaluation revealed elevated hemoglobin and hematocrit. His serum glucose level was 88 mg/dL (reference range: 74-99 mg/dl), and urine drug screening was positive for cannabinoids. A CT angiography (CTA) of the head and neck ruled out large vessel occlusion, aneurysm, or any other vascular abnormalities. A CT venography was obtained to evaluate the cause of the SAH, which demonstrated dural venous sinus thrombosis involving the mid-to-posterior segment of the superior sagittal sinus (Figure 2) and the right transverse sinus. The SAH in the left frontoparietal region was redemonstrated on venous imaging. 

The patient was admitted to the neurocritical care unit, and he was started on a therapeutic intravenous heparin and levetiracetam for seizures. A pro-thrombotic study was initiated, and hematology-oncology was consulted for underlying thrombophilia. On the second day of admission, he had another episode of focal seizure involving the right lower extremity, so we increased the levetiracetam dose and added intravenous fosphenytoin.

Pro-thrombotic workup was unremarkable (Table 1). The JAK2 V617F mutation was negative, and the serum erythropoietin level was normal. Protein C activity was mildly reduced (73%), likely due to the acute phase of illness. Serial hemoglobin and hematocrit are shown in Table 2.

**Table 1:** Hypercoagulable workup performed during hospitalization

Laboratory parameters	Values	Reference range
Factor V activity (%)	113	50-150
Factor VIII activity (%)	145	50-150
Anti-thrombin III activity (%)	85	83-122
Protein C activity (%)	73	>80
Protein C antigen (%)	70	63-153%
Protein S antigen, free (%)	89	74-147
Protein S antigen, total (%)	109	84-134
Prothrombin mutation	Negative	-
Lupus anticoagulant	Negative	-
Anti-cardiolipin IgG (GPL)	1.8	0.0-10
Anti-cardiolipin IgM (MPL)	1.2	0.0-10.0

**Table 2:** Trend of hematological parameters during hospitalization

Laboratory parameters	Day 1	Day 2	Day 3	Day 4	Reference range
Hemoglobin (g/dl)	19.5	17.4	15.5	16.9	13-17
Hematocrit (%)	61	55.6	48.7	53	40.7-50.3
White blood cell count (k/uL)	20.0	12.8	13.4	9.9	3.5-11.3
Platelet count (k/uL)	258	204	170	183	138-453

After the exclusion of secondary causes of polycythemia, testosterone-induced erythrocytosis was determined as the underlying etiology for CVST. The patient was discharged with instructions to maintain anticoagulation (apixaban 5 mg twice a day), antiepileptics, and stop testosterone therapy. He was advised to follow up with neurology, hematology, endocrinology, endovascular specialists, and his primary care provider. Polysomnography was also recommended to evaluate potential obstructive sleep apnea as a contributing factor to secondary polycythemia.

## Discussion

Testosterone therapy is associated with erythrocytosis and increases the risk of thromboembolic complications, including DVT and PE, and rarely CVST [1,2]. Polycythemia is an increase by >16.5 g/dl hemoglobin or >49% hematocrit in men, and >16 g/dl hemoglobin or >48% hematocrit in women. Testosterone increases red blood cell mass by stimulating erythropoiesis, often leading to >18.5 g/dL hemoglobin levels and >52% hematocrit [4]. Testosterone significantly increases hematocrit. It causes polycythemia by increasing the set point of erythropoietin to higher hemoglobin levels and increasing iron bioavailability by decreasing ferritin and hepcidin levels [5]. When persistent, this can increase blood viscosity and predispose to venous thrombosis.

Polycythemia vera was ruled out in this patient by negative JAK2 testing and normal erythropoietin levels. Other causes of secondary polycythemia, like chronic obstructive pulmonary disease (COPD), congenital heart diseases, and tumors, were ruled out through clinical evaluation and diagnostic testing, establishing the diagnosis of secondary polycythemia from testosterone use, a known risk associated with androgen use [2,4].

Given the established risk of erythrocytosis with testosterone therapy, current guidelines recommend regular monitoring of hematocrit and hemoglobin every three to six months during the first year of treatment and annually after. Therapy should be adjusted or paused if hematocrit exceeds 54%, as this significantly increases thrombotic risk. If the hematocrit normalizes, a lower dose of testosterone therapy should be initiated. If the hematocrit remains elevated with the lower testosterone dose, the patient should be evaluated for a treatable cause, like obstructive sleep apnea. If no treatable cause is identified, phlebotomy can be considered [6]. 

Surveillance is often underutilized in practice despite the recommendations, leading to delayed recognition of hematologic complications. This case emphasizes the need for proactive hematologic monitoring and individualized risk assessment in patients on long-term testosterone therapy. Recognizing CVST as a thromboembolic complication of testosterone use is essential for prompt management. 

Seizures in CVST are common and are linked to venous infarction, increased intracranial pressure, or hemorrhagic transformation [3]. Subarachnoid hemorrhage in CVST, although rare, typically results from the rupture of engorged cortical veins under venous hypertensive stress [7]. Management includes prompt anticoagulation, even in the presence of SAH, due to the underlying thrombotic mechanism [8]. Our patient responded well to intravenous heparin, followed by direct oral anticoagulation. Antiepileptic therapy was initiated in this patient based on seizure semiology and hemorrhagic findings.

Clinicians must remain vigilant for CVST and other thrombotic complications in patients using anabolic steroids or testosterone, particularly when laboratory values suggest erythrocytosis. The risk of venous thromboembolism is directly related to the level of hematocrit [9]. Discontinuation of the causative agent and anticoagulation may be necessary. 

## Conclusions

This case report highlights the life-threatening consequences of testosterone-induced erythrocytosis, including CVST. So, an early recognition with prompt anticoagulation and discontinuation of the causative agent is crucial for a better prognosis. As testosterone therapy becomes increasingly common, this report reminds us of the importance of hematological surveillance in these patients due to thrombotic risk. This case adds to the growing awareness of exogenous androgen risks and highlights the need for vigilance in patients with neurologic symptoms and elevated hematologic indices.

## References

[1] Glueck CJ, Richardson-Royer C, Schultz R, Burger T, Bowe D, Padda J, Wang P (2014). Testosterone therapy, thrombophilia-hypofibrinolysis, and hospitalization for deep venous thrombosis-pulmonary embolus: an exploratory, hypothesis-generating study. Clin Appl Thromb Hemost.

[2] Ohlander SJ, Varghese B, Pastuszak AW (2018). Erythrocytosis following testosterone therapy. Sex Med Rev.

[3] Ferro JM, Canhão P, Stam J, Bousser MG, Barinagarrementeria F (2004). Prognosis of cerebral vein and dural sinus thrombosis: results of the International Study on Cerebral Vein and Dural Sinus Thrombosis (ISCVT). Stroke.

[4] Bachman E, Travison TG, Basaria S (2014). Testosterone induces erythrocytosis via increased erythropoietin and suppressed hepcidin: evidence for a new erythropoietin/hemoglobin set point. J Gerontol A Biol Sci Med Sci.

[5] Panjwani A, Burle VS, Raj R (2023). Secondary polycythemia and venous thromboembolism: a systematic review. F1000Research.

[6] Bhasin S, Brito JP, Cunningham GR (2018). Testosterone therapy in men with hypogonadism: an Endocrine Society clinical practice guideline. J Clin Endocrinol Metab.

[7] Saposnik G, Barinagarrementeria F, Brown RD Jr (2011). Diagnosis and management of cerebral venous thrombosis: a statement for healthcare professionals from the American Heart Association/American Stroke Association. Stroke.

[8] Coutinho JM, Ferro JM, Canhão P, Barinagarrementeria F, Bousser MG, Stam J (2010). Unfractionated or low-molecular weight heparin for the treatment of cerebral venous thrombosis. Stroke.

[9] Braekkan SK, Mathiesen EB, Njølstad I, Wilsgaard T, Hansen JB (2010). Hematocrit and risk of venous thromboembolism in a general population. The Tromsø study. Haematologica.

